# A Case of Isolated Abnormal Behavior Associated With Vitamin B12 Deficiency

**DOI:** 10.7759/cureus.115632

**Published:** 2026-09-02

**Authors:** Samar A Abdelaziz Ibrahim, Sara M Ahmed Mohamed, Sara A Al Quradaghi, Sarah A Abdallah Ali, Hana Nishan, Minhaj Mammadu, Nedia Neffati, Nishan K Purayil

**Affiliations:** 1 Medical Education Department, Hamad General Hospital, Hamad Medical Corporation, Doha, QAT; 2 Surgery Department, Almanagil Teaching Hospital, Almanagil, SDN; 3 General Internal Medicine Department, Hamad General Hospital, Hamad Medical Corporation, Doha, QAT; 4 Clinical Medicine Department, Weill Cornell Medicine - Qatar, Doha, QAT; 5 Clinical Department, College of Medicine-QU Health, Qatar University, Doha, QAT

**Keywords:** cyanocobalamin, neuropsychiatric, psychosis, serum vitamin b12, vitamin b12 megaloblastic anemia

## Abstract

Vitamin B12 is a water-soluble nutrient found mainly in animal-derived foods. Vitamin B12 deficiency is a common condition, presenting with hematologic and neurologic abnormalities, but rarely presents as isolated psychosis in a young adult. A 28-year-old male patient was brought in for severe agitation and confusion. Laboratory evaluation revealed severe vitamin B12 deficiency (serum vitamin B12 of <73.8 pmol/L) with elevated homocysteine (25.4 umol/L) and methylmalonic acid (0.95 umol/L) levels. The patient received vitamin B12 replacement, after which his confusion and behavioral disturbances improved significantly, and further psychiatric evaluation failed to reveal a primary psychotic or mood disorder. This case highlights the importance of considering vitamin B12 deficiency in patients with isolated neuropsychiatric symptoms, even in the absence of typical macrocytosis, and demonstrates the potential for significant clinical recovery following timely treatment with vitamin supplementation.

## Introduction

Vitamin B12 is a water-soluble vitamin primarily obtained through the consumption of animal products, including red meat, dairy products, and eggs. Following absorption, vitamin B12 serves as a critical enzymatic cofactor in the synthesis of deoxyribonucleic acid, fatty acids, and myelin. As a result, inadequate levels can lead to various hematologic and neurologic symptoms [1]. While global vitamin B12 deficiency remains most prevalent in populations with low socioeconomic status, it is also a significant health concern in industrialized nations, where it disproportionately affects the elderly [2].

Vitamin B12 deficiency classically presents with hematological and neurological manifestations. Hematological findings include megaloblastic anemia and, in severe cases, pancytopenia, while neurological complications range from peripheral neuropathy and paresthesia to subacute combined degeneration of the spinal cord [3].

In addition to its well-recognized hematological and neurological manifestations, vitamin B12 deficiency has been associated with a wide spectrum of neuropsychiatric symptoms, including depression, cognitive impairment, psychosis, delirium, and behavioral disturbances. Notably, these manifestations may occur in the absence of hematological abnormalities, making diagnosis particularly challenging. As a result, patients may undergo extensive neurological and psychiatric investigations before the underlying deficiency is identified [3].

We report the case of a young adult man who presented with acute neuropsychiatric and behavioral disturbances, prompting comprehensive neurological and psychiatric evaluation.

## Case presentation

A 28-year-old male patient, an expatriate worker in Qatar, was brought to the emergency department for psychiatric assessment for abnormal behavior. He was found by police wandering the streets, confused and agitated.

His past medical and surgical history was unremarkable. He denied alcohol and illicit drug use, and his family history was unknown for psychiatric disorders. He was previously fully independent, ambulatory, and communicative.

The patient reported that he had been experiencing homesickness since arriving in Qatar a month and a half ago, with increasing distress over the preceding week. He described social withdrawal and workplace interpersonal conflicts, leading to the authorities' involvement. He denied any prior head trauma before admission. He also denied auditory or visual hallucinations, suicidal or homicidal ideation, or a history of psychiatric illness.

During the early course of admission, he required sedation due to severe agitation and aggressive behavior. While in the emergency department, he hit his head against a glass door. Over the subsequent 48 hours, his agitation resolved; however, low mood and depressive symptoms were noted.

Examination and initial investigations

On initial assessment in the emergency department, the patient was hemodynamically stable apart from sinus tachycardia. Vital signs were as follows: heart rate 114 beats per minute, respiratory rate 19 breaths per minute, blood pressure 124/70 mmHg, and oxygen saturation 98% on room air.

On examination, he was alert and oriented, with a Glasgow Coma Scale score of 15/15. However, he was agitated and required chemical restraint. Pupils were equal and reactive bilaterally, and full motor strength was observed in all limbs with no focal neurological deficits. Examination revealed periorbital bruising around the left eye, dried blood around the right ear, and bilateral wrist swelling and tenderness. Cardiovascular, respiratory, and abdominal examinations were unremarkable. Initial psychiatric assessment was inconclusive as he was sedated. On repeat assessment later during his admission, his mental status exam was unremarkable with normal and appropriate appearance, behavior and speech, euthymic mood with congruent affect, intact perception, thought process and content without hallucinations or delusions, intact cognition, and memory with preserved insight and good judgment.

Initial laboratory investigations demonstrated leukocytosis, elevated C-reactive protein, and a mildly prolonged prothrombin time. Biochemical analysis showed low adjusted calcium levels and elevated aspartate aminotransferase, creatine kinase, and serum myoglobin levels. Nutritional markers showed serum vitamin B12 of 73.8 pmol/L, with elevated homocysteine and methylmalonic acid levels (Table 1). Serum ethanol level was normal. However, detailed toxicology evaluation for illicit drugs and other prescription and nonprescription substances was not performed.

**Table 1:** Summary of patient’s routine blood test results at the time of initial presentation in the emergency department

Parameter	Result	Reference range	Units
White blood cell count	22.6	4-10	×10^3^/μL
Hemoglobin	15.4	13-17	g/dL
Mean corpuscular volume	95.1	83.0-101.1	fL
Platelet	437	150-410	×10^3^/μL
C-reactive protein	6.7	0.0-5	mg/L
Vitamin B12	<73.8	145-569	pmol/L
Folate	13	10-70	nmol/L
Homocysteine	25.4	0.0-12.0	umol/L
Methylmalonic acid	0.95	0.0-0.40	umol/L
Prothrombin time	14.1	9.4-12.5	seconds
International normalized ratio	1.3	0.8-1.2	-
Creatine kinase	>22,000	39-308	U/L
Myoglobin	10,537	28-72	ng/mL
Aspartate aminotransferase	144	0-40	U/L
Alanine aminotransferase	39	0-41	U/L
Alkaline phosphatase	86	40-129	U/L
Urea	4.9	2.5-7.8	mmol/L
Creatinine	99	62-106	umol/L
Sodium	139	133-146	mmol/L
potassium	3.7	3.5-5.3	mmol/L
Calcium	2.25	2.12-2.62	mmol/L
Adjusted calcium	2.17	2.20-2.60	mmol/L
Total protein	78	60-80	g/L
Albumin	44	35-50	g/L
Ethanol	2.2	0-44.9	mmol/L
Glucose	6.2	3.3-5.5	mmol/L

Diagnostic workup

The patient underwent urgent neuroimaging following presentation with agitation and head trauma. Noncontrast computed tomography (CT) scan of the head performed in the emergency department on the day of presentation demonstrated a left frontal subarachnoid hemorrhage (Figure 1).

**Figure 1:**
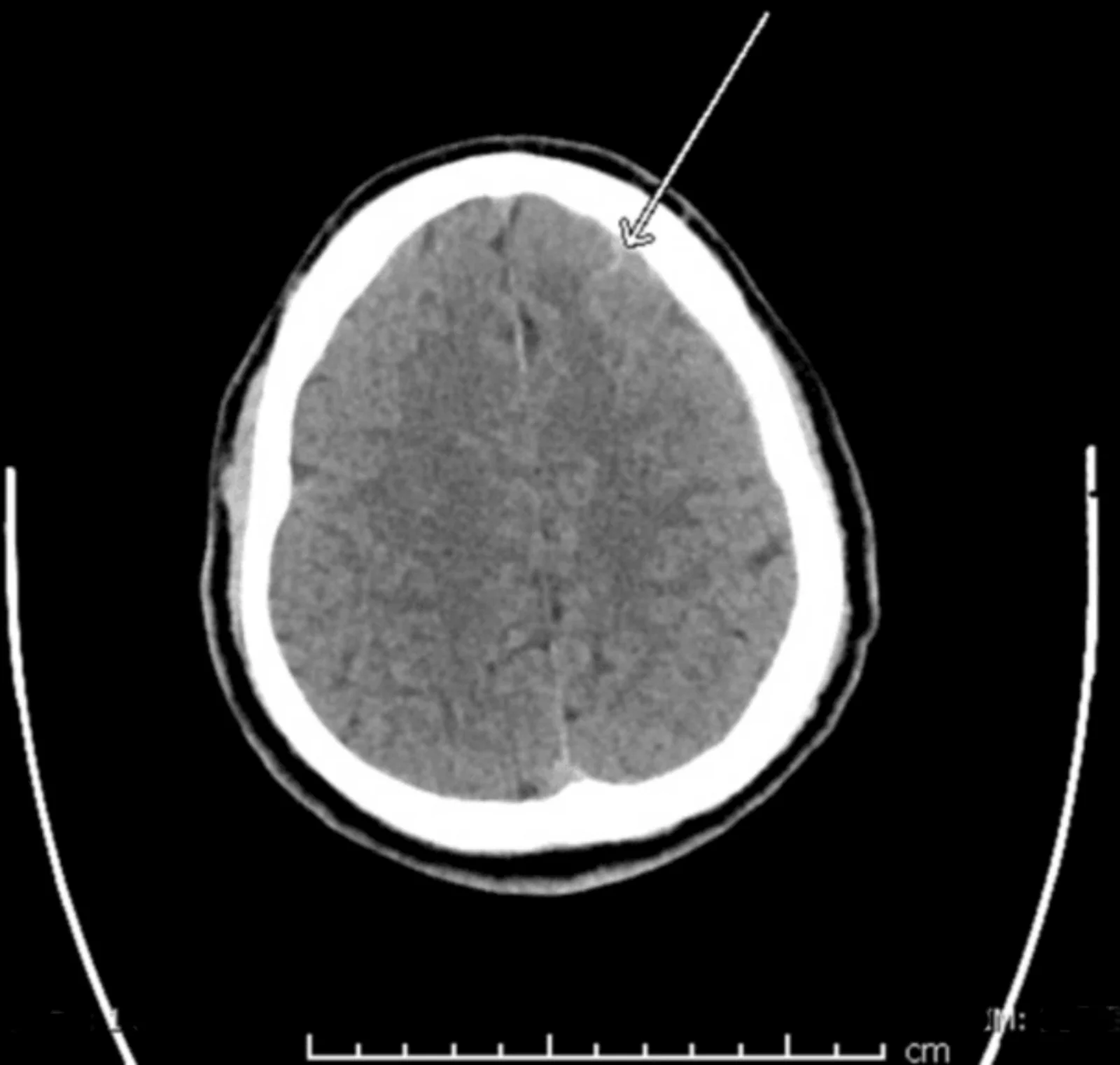
Noncontrast CT head showing subarachnoid hemorrhage (white arrow) in the left frontal lobe CT: computed tomography

Additional imaging, including CT of the face and radiographs of the pelvis, femur, and both wrists, revealed no evidence of fractures. Further evaluation included an MRI of the brain and an electroencephalogram (EEG). MRI of the head with contrast, performed 48 hours after presentation, demonstrated sulcal T2 hyperintensity in the left frontal lobe (Figure 2) with diffuse pachymeningeal enhancement (Figure 3), most consistent with traumatic changes following in-hospital head injury. EEG revealed mild generalized slowing without epileptiform activity.

**Figure 2:**
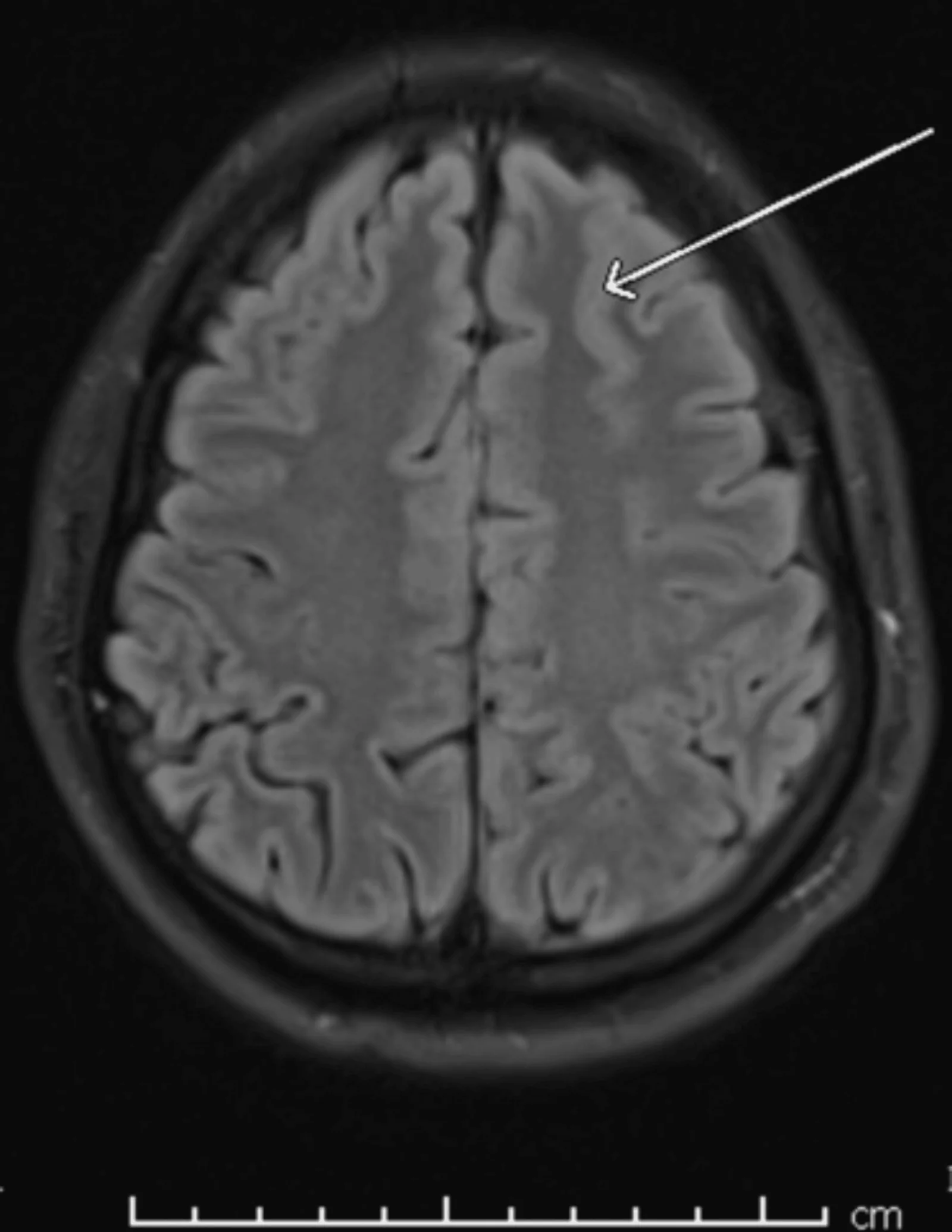
Contrast head MRI showing sulcal T2 hyperintensity in the left frontal lobe on FLAIR images (white arrow) FLAIR: fluid-attenuated inversion recovery

**Figure 3:**
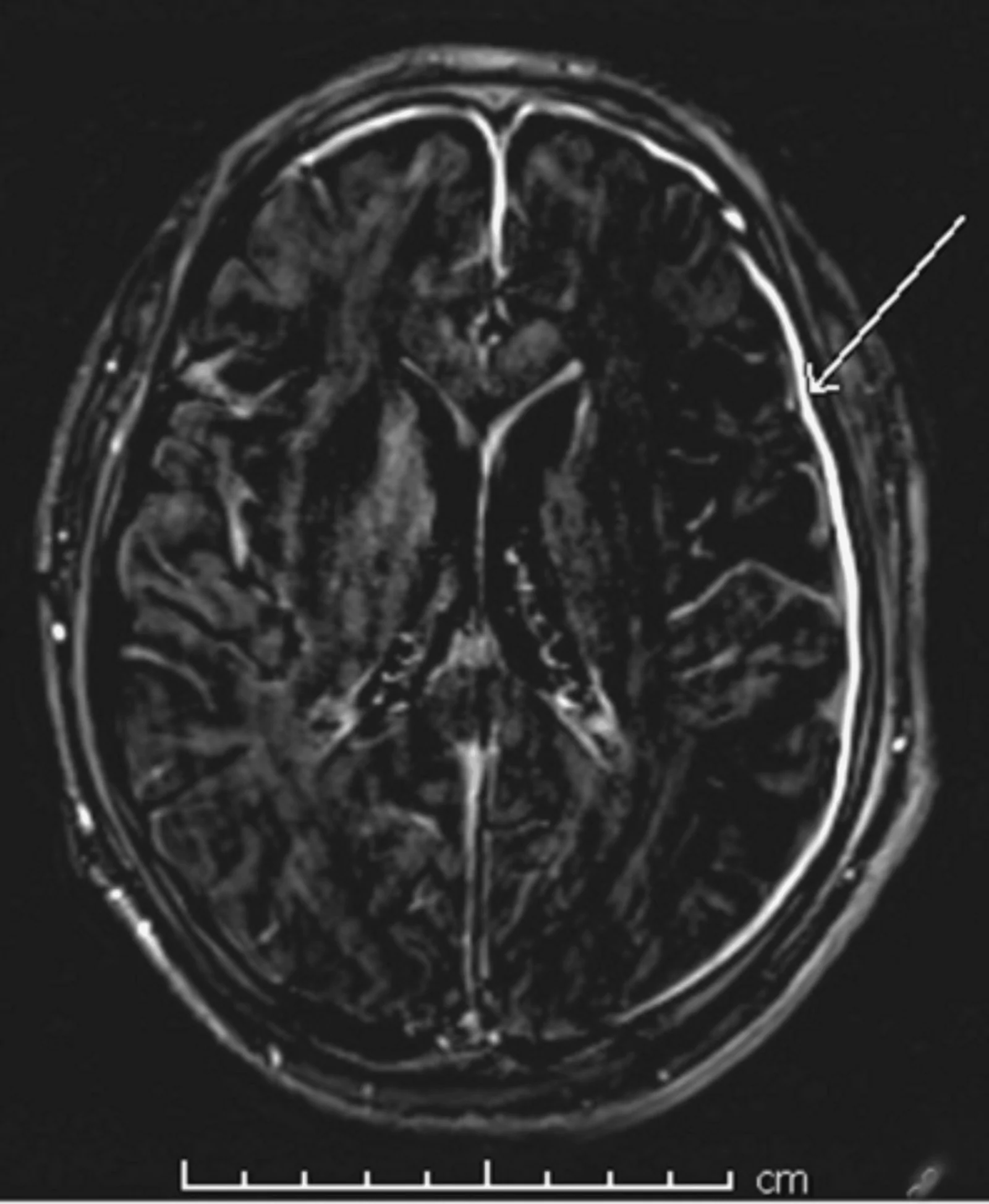
Contrast head MRI showing diffuse pachymeningeal enhancement (white arrow)

A lumbar puncture was performed to exclude an infectious etiology. Cerebrospinal fluid (CSF) analysis was clear and colorless, with the presence of red blood cells, likely secondary to the known traumatic subarachnoid hemorrhage. CSF bacterial culture, cryptococcal antigen, fungal studies, and viral polymerase chain reaction panel were all negative (Table 2). Extensive autoimmune and inflammatory investigations are shown in Table 3.

**Table 2:** Summary of the patient’s cerebrospinal fluid analysis at the time of admission IgG: immunoglobulin G

Parameter	Result	Units
Color	Colorless	-
Appearance	Clear	-
Red blood cell	149	μL
Total nucleated cells	2	μL
Glucose	3.99	mmol/L
Protein	0.35	g/L
Albumin	227	mg/L
IgG	30	mg/L
IgG index	0.3	-
IgG oligoclonal bands	Not detected	-
Bacterial culture	Negative	-
Cryptococcal culture	Negative	-

**Table 3:** Summary of the patient’s autoimmune, immunology, and infectious panel results IgG: immunoglobulin G; IgM: immunoglobulin G; CSF: cerebrospinal fluid; PCR: polymerase chain reaction; ANA CTD: antinuclear antibody connective tissue disease

Parameter	Result	Reference range/interpretation	Units
Rheumatoid factor	13	0-14	IU/mL
Antiparietal cell antibody	Negative	Negative	-
Antineutrophil cytoplasmic antibody	Negative	Negative	-
ANA CTD	Negative	Negative	-
Anticardiolipin IgG antibody	>0.50	Negative	GPL
Anticardiolipin IgM antibody	6.40	Negative	MPL
Lupus anticoagulant	>0.4	Negative	u/mL
Anti-SS-A (Ro) antibody	>0.6	Negative	u/mL
Antithyroid peroxidase	15	0-34	IU/mL
Complement C3	0.99	0.90-1.80	g/L
Complement C4	0.21	0.10-0.40	g/L
CD3	59.96	57.52-83.11	%
CD3 absolute count	797.00	856.00-2,669.00	cells/mcL
CD3+/CD4+	39.96	31.45-62.38	%
CD3+/CD4+ absolute count	531.00	491.00-1,734.00	cells/mcL
CD3+/CD8+	16.48	9.55-38.32	%
CD3+/CD8+ absolute count	219.00	162.00-1,074.00	cells/mcL
CD19	22.28	5.89-24.21	%
CD19 absolute count	296.00	73.00-562.00	cells/mcL
CD3-/CD16+/CD56+	17.20	5.17-30.36	%
CD3-/CD16+/CD56+ absolute count	229.00	108.00-680.00	cells/mcL
CD4:CD8 ratio	2.41	1.0-4.0	-
Blood culture (aerobic)	Negative	Negative	-
Blood culture (anaerobic)	Negative	Negative	-
Fungal culture	Negative	Negative	-
CSF multiplex PCR panel			
Enterovirus	Negative	Negative	-
Cytomegalovirus	Negative	Negative	-
Parechovirus	Negative	Negative	-
Human herpesvirus 6	Negative	Negative	-
Herpes simplex virus 1	Negative	Negative	-
Herpes simplex virus 2	Negative	Negative	-
Varicella zoster virus	Negative	Negative	-
*Escherichia coli* K1	Negative	Negative	-
Haemophilus influenzae	Negative	Negative	-
Listeria monocytogenes	Negative	Negative	-
Neisseria meningitidis	Negative	Negative	-
Streptococcus pneumoniae	Negative	Negative	-
Streptococcus agalactiae	Negative	Negative	-
Cryptococcus neoformans/Cryptococcus gattii	Negative	Negative	-

Overall, investigations confirmed traumatic subarachnoid hemorrhage, rhabdomyolysis, and severe vitamin B12 deficiency, without evidence of infectious, autoimmune, or primary structural neurological disease beyond trauma.

Management and outcome

Following initial stabilization, targeted metabolic therapeutic intervention was initiated for the severe cobalamin deficiency. The patient received a loading regimen of intramuscular vitamin B12 (cyanocobalamin) 1,000 mcg once daily for five consecutive days, followed by a transition to high-dose oral therapy consisting of 2,000 mcg daily for an additional five days. He was subsequently managed supportively with intravenous fluids and thiamine, with close monitoring of rhabdomyolysis markers, and his condition gradually improved during admission. Given the remote possibility of seizure with post-ictal state as a differential and new traumatic subarachnoid hemorrhage, valproate 500 mg twice daily was initiated. Over the following days, confusion and behavioral disturbance improved significantly, and the patient was deemed medically stable for transfer to psychiatric care.

During psychiatric admission, serial assessments did not identify evidence of a primary psychotic or mood disorder. The patient remained calm, coherent, and behaviorally stable. Residual anxiety symptoms were managed with escitalopram and short-term clonazepam. Following resolution of neuropsychiatric symptoms, he was discharged in stable condition with a plan to continue vitamin therapy with outpatient neurology follow-up to taper and discontinue valproate.

## Discussion

This case describes a young adult man who presented with acute agitation, confusion, and disorganized behavior, prompting a broad neurological and psychiatric evaluation. His presentation was complicated by traumatic subarachnoid hemorrhage during agitation in the emergency department and by psychosocial stressors related to recent relocation and occupational difficulties. Extensive investigations for infectious, structural, autoimmune, and toxic causes were unyielding, except for markers suggestive of vitamin B12 deficiency. Notably, classical hematological manifestations were absent, and the diagnosis was established through biochemical evidence of deficiency and associated metabolic markers. Clinical improvement following vitamin B12 replacement supports a contributory role of cobalamin deficiency in the patient’s neuropsychiatric presentation. Overall, this case highlights the diagnostic complexity of acute neuropsychiatric presentations and underscores the importance of considering vitamin B12 deficiency even when initial findings suggest alternative neurological or psychiatric etiologies. Vitamin B12 deficiency is associated with a broad spectrum of neuropsychiatric manifestations, including mood changes, psychosis, agitation, cognitive impairment, and delirium. These features may occur in isolation and mimic primary psychiatric disorders, leading to misdiagnosis and delayed recognition of the underlying deficiency. Case reports and series show that psychiatric symptoms may be the predominant or sole presentation, with improvement following vitamin B12 replacement therapy [4-7]. This underscores the need to consider vitamin B12 deficiency in acute psychiatric presentations without an obvious primary etiology.

A key challenge in vitamin B12 deficiency is the frequent absence of macrocytic anemia or other hematological abnormalities at presentation. Neuropsychiatric symptoms may precede or occur independently of hematological changes, making reliance on routine blood indices insufficient for diagnosis. Functional markers such as homocysteine and methylmalonic acid are therefore more sensitive in atypical cases [6,7]. This diagnostic gap often leads to initial misattribution to primary psychiatric or neurological conditions, as seen in our patient, whose presentation initially misdirected the diagnostic focus toward a primary psychiatric breakthrough or acute trauma before metabolic screening was performed.

The neuropsychiatric manifestations of vitamin B12 deficiency are thought to result from disruption of multiple biochemical pathways essential for central nervous system function. Vitamin B12 and folate act as cofactors in one-carbon metabolism, particularly in the conversion of homocysteine to methionine, which is required for the synthesis of S-adenosylmethionine, a key methyl donor involved in neurotransmitter metabolism, DNA regulation, and phospholipid synthesis [8]. Impairment of this pathway leads to defective methylation processes, affecting myelin maintenance and neuronal integrity. In addition, vitamin B12 deficiency leads to accumulation of methylmalonic acid and homocysteine, both of which have been implicated in neurotoxicity and demyelination [3,6]. These biochemical disturbances are associated with a range of neuropsychiatric manifestations, including mood disorders, psychosis, delirium, and cognitive impairment [6-8]. Notably, these metabolic abnormalities may precede hematological changes, providing a plausible explanation for isolated neuropsychiatric presentations, as observed in the present case.

This case is consistent with previous reports of vitamin B12 deficiency presenting with acute neuropsychiatric symptoms that improve following replacement therapy [4-6]. However, diagnostic evaluation was complicated by traumatic subarachnoid hemorrhage, acute agitation requiring sedation, and psychosocial stressors, which broadened the initial differential diagnosis. Despite these confounders, biochemical testing confirmed severe vitamin B12 deficiency with elevated homocysteine and methylmalonic acid, and the patient showed marked clinical recovery after treatment. This highlights the importance of considering vitamin B12 deficiency even in complex clinical settings with multiple overlapping factors.

Vitamin B12 deficiency should be considered in patients presenting with acute neuropsychiatric symptoms, even in the absence of classical hematological abnormalities. Delayed recognition may prolong diagnostic evaluation and increase the risk of persistent neurological dysfunction. Early screening using serum vitamin B12 and functional biomarkers such as homocysteine and methylmalonic acid is essential. Prompt treatment is associated with significant clinical improvement and favorable outcomes in reversible cases.

## Conclusions

This case demonstrates that severe vitamin B12 deficiency should be considered in patients who present with acute isolated neuropsychiatric and behavioral disturbances, even in the absence of classical macrocytic anemia or other hematological abnormalities. The diagnostic process may be challenging in acute care settings, particularly when confounded by psychosocial stressors or concurrent traumatic neurological injury. Clinicians should maintain a high index of suspicion and consider early measurement of functional biomarkers such as homocysteine and methylmalonic acid in patients with unexplained first-episode psychiatric or behavioral presentations. As vitamin B12 deficiency is a reversible and treatable cause of neuropsychiatric dysfunction, early recognition and prompt treatment are essential to ensure recovery and prevent potential long-term neurological sequelae.
